# Psychiatric morbidity among men using anabolic steroids

**DOI:** 10.1002/da.23287

**Published:** 2022-10-25

**Authors:** Josefine Windfeld‐Mathiasen, Thea Christoffersen, Niels August Willer Strand, Kim Dalhoff, Jon Trærup Andersen, Henrik Horwitz

**Affiliations:** ^1^ Department of Clinical Pharmacology Bispebjerg and Frederiksberg Hospital Copenhagen Denmark; ^2^ Department of Clinical Medicine University of Copenhagen Copenhagen Denmark

**Keywords:** androgenic anabolic steroids, depression, depressive adverse reactions, neuropsychiatric effects

## Abstract

**Objective:**

The purpose of this study was to investigate the psychiatric morbidity among men with abuse of anabolic steroids.

**Methods:**

The design is a retrospectively matched cohort study. Five hundred and fourty‐five males, who tested positive for anabolic steroids in Danish fitness centers during the period January 3, 2006 to March 1, 2018, were matched with 5450 randomly chosen male controls. Data was cross‐referenced with seven national registers pertaining to information about education, employment status, and psychiatric comorbidity. Main outcomes and measures were prescription of psychopharmacological treatment.

**Results:**

The incidence of treatment with anxiolytics (HR: 2.34, 95% CI: 1.62−3.38) and antipsychotics (HR: 2.69, 95% CI: 1.99−3.63) displayed a remarkable increase in the years following doping sanction, compared to the control group. The prevalence of antidepressant use was already markedly elevated several years before doping sanction, but also displayed a higher incidence in the years following sanction (HR: 1.65, 95% CI: 1.28−2.13). The associations remained highly significant after controlling for socioeconomic factors.

**Conclusion:**

Anabolic steroids use is strongly associated with psychiatric morbidity.

## 1 INTRODUCTION

The prevalence of anabolic androgenic steroid (AAS) use amongst young men is reported as high as 6% (Sagoe et al., 2014). Physical strength is usually perceived as markers of masculinity, health, and high genetic quality, (Sell et al., 2017) thus allegedly creating an incentive to use AAS. Furthermore, users of AAS have been found to have a high degree of a dysmorphic body image before initiating the use of AAS (Smit et al., 2021).

It is well known that AAS causes somatic adverse effects such as hypogonadism, gynecomastia, acne, and infertility (Horwitz, Andersen & Dalhoff, 2019). Furthermore, an additional number of psychiatric adverse effects such as aggressiveness, depression, and mania has been reported (Amaral et al., 2022; Pope et al., 2014). A recent study by Nackeeran et al. (2022) has shown that testosterone use is associated with a higher risk of major depressive disorders and suicide attempt.

AAS affects the hypothalamic‐pituitary‐gonadal axis and, consequently, exogen administration of AAS decreases the endogenous production of follicle stimulating hormone, luteinizing hormone, and testosterone (Rahnema et al., 2014). Recent publications suggest another link between testosterone and mental health; first, testosterone replacement therapy in hypogonadal men has been seen to improve psychological well‐being; second, androgen deprivation therapy in men with prostate cancer is associated with depression (Fischer et al., 2019; Hartgens & Kuipers, 2004; McHenry et al., 2014; Nead, 2019; Walther et al., 2019). Third, a recent study conducted by Rasmussen et al. (2016) found that former AAS users exhibited significantly lower plasma testosterone levels and also exhibited a higher proportion of depressive symptoms (no correlation test reported).

AAS is suspected to induce alterations in central nervous system structure and function which has been explored in several studies. Long‐term use of AAS was found to be associated with amygdala enlargement and poorer visual spatial function in AAS using male weightlifters compared to weightlifters with no experience with AAS (Kaufman et al., 2015). With a similar design, Bjørnebekk et al. (2017) found that AAS users had a thinner cortex in various regions as well as reduced total gray matter, cerebral cortex, and putamen.

Altogether, this suggests that chronic use of AAS is associated with detrimental mental health effects, and the current study aims to uncover the neuropsychiatric adverse effects associated with AAS.

## 2 MATERIALS AND METHODS

### 2.1 Research endpoints

1. To compare the incidence and prevalence of psychopharmacological treatment in AAS users with that of a cohort of age and gender matched controls.
2. To compare the prevalence of psychiatric diagnoses in AAS users with that of a cohort of age and gender matched controls.

### 2.2 Design

Retrospectively matched cohort study.

### 2.3 The anti‐doping program

As previously described (Christoffersen et al., 2019; Horwitz, Andersen & Dalhoff, 2019; Windfeld‐Mathiasen et al., 2021), it has long been a political ambition to create a safe and clean fitness environment in Denmark free of illegal doping. In Denmark 342 fitness centers collaborated with Anti Doping Denmark hereby covering 80% of all fitness center members. Anti Doping Denmark conducted around 1000 inspections of these centers annually and tested random athletes in the participating centers for use of illegal doping drugs. In the first 2 years doping controls were conducted on a broader range of fitness members, however, this testing strategy was not cost effective and based on the experiences the testing was afterwards targeted subjects with an AAS abuser phenotype. Thus, the doping controls have primarily been targeted at persons visually suspected of AAS use, in other words muscular men and women who engage in weightlifting. The time from test to sanction was approximately 3 to 4 weeks. Subjects were tested against a specific doping list developed and maintained by the Danish Medicines Agency, and the analysis were performed by a World Anti‐Doping Agency certified laboratory.

In less than 1% of the cases, subjects tested positive for testosterone alone. In all other cases, subjects test positive for exogenous anabolic steroids, which are not approved by the Danish Medicines Agency. In those rare cases where subjects test positive for testosterone alone, and hence could be the result of treatment by a physician, the subject were allowed to submit documentation for the treatment and, consequently, has been excluded from the cohort. For a description of the sanctioned subjects pretest morbidity please see (Horwitz, Andersen & Dalhoff, 2019). A positive test or refusal to participate in the doping test resulted in a doping sanction which meant exclusion from all fitness centers in collaboration with Anti Doping Denmark for 2 years, and exclusion from all organized sports for 4 years. The sanctioned individual was registered in a national doping register, thus ensuring that only people with a clean profile could get access to fitness centers as well as sports clubs.

### 2.4 Cohorts

From January 3, 2006 to March 1, 2018, 545 men were sanctioned due to AAS traces in the urine sample provided, and 644 men were sanctioned following their refusal to deliver a urine sample. The primary analyses are based solely on data from the 545 laboratory‐confirmed AAS users (AAS‐user cohort), while the data from the cohort without urine samples is used as a replication cohort and can be found in Supporting Information data. For each AAS user, we randomly chose 10 controls from the general population, matched by age and gender from the same birth cohort living in Denmark at the time (control cohort).

### 2.5 Registries

All residents in Denmark have a specific social identification number (CPR) used in all interactions with the state and the healthcare system. In this paper, we cross‐referenced the patients' CPR numbers from AAS users with data from the Danish Civil Registration System, The DREAM database, The Danish National Registry of Patients, The National Hospital Register, the Danish Education Registers, The Danish National Prescription Register, and the Danish Psychiatric Central Research Registry as performed in our former research papers (Christoffersen et al., 2019; Horwitz, Andersen & Dalhoff, 2019; Windfeld‐Mathiasen et al., 2021). A detailed description of the registers can be found in Supporting Information data. Patients were followed until May 16, 2018.

### 2.6 Methods

We generated a retrospectively matched cohort data set and analyzed it using several methods. The 545 males who tested positive for AAS in Danish fitness centers during the period January 3, 2006 to March 1, 2018, were matched with 5450 male controls. We cross‐referenced their personal identification numbers with seven Danish national registries related to health information. We followed this cohort from 10 years before baseline and until the end of follow‐up in May 2018. We defined chronic use of medication as five or more redeemed prescriptions.

### 2.7 Statistics

Data was tested using the *t*‐test for approximately normally distributed continuous variables, and *χ* ^2^ test for categorical variables.

The cohorts were followed from 10 years before baseline and until the end of follow‐up in May 16, 2018. In our calculation of observation time, we accounted for migration in and out of Denmark. All statistics were computed in SAS 9.4.

In our statistical analysis, we used Poisson regression, Cox proportional hazards regression, and logistic regression models. The Poisson regression model and Cox proportional hazards regression were used in relation to the length of follow‐up, primarily to investigate the incidence rates. Pertaining to the prevalence of chronic outcomes we used the logistic regression model. Our data was already matched on age and gender, and we furthermore adjusted for potential confounders known at baseline; country of origin, employment status, and education (see Table 1).

**Table 1:** Baseline characteristics

		AAS users	Control	*p* Value
Age (years)	Mean (SD)	26.2 (6.3)	26.2 (6.3)	
Education groups		2.2	5.1	<.0001^a^
Missing	**%**			
10 years or less	**%**	48.8	35.5	
10−12 years	**%**	9.9	22.0	
12−15 years	**%**	36.5	24.6	
15 years or more	**%**	2.6	12.8	
Country of origin				.22
Danish	**%**	85.5	84.3	
Immigrants	**%**	10.6	12.7	
Descendants of immigrants	**%**	3.9	3.0	
Occupational status				<.0001^a^
Missing	**%**	1.7	1.1	
Self‐supporting	**%**	78.9	85.9	
Sick leave—temporarily	**%**	2.4	1.7	
Disability benefits	**%**	2.2	2.3	
Unemployed	**%**	14.9	9.1	
Complete data	**%**	96.5	94.6	.06

Abbreviations: AAS, anabolic androgenic steroid; SD, standard deviation.

^a^ Significant *p* value obtained from ordinary *χ* ^2^ table test.

As sensitivity analysis we performed a self‐controlled observational design as we assumed that individuals who were just about to test positive for AAS use and individuals who had just been tested positive, did not differ substantially except through the effect of the doping sanction and possible abstinence from AAS. We therefore restrict the sample to a narrow time window consisting of the 2 years leading up to the positive test and the 2 years that followed.

## 3 RESULTS

### 3.1 Baseline characteristics

We followed 545 male AAS users and 5450 age and gender matched controls, from 10 years before doping sanction/baseline and until May 16, 2018. The average age at the time of doping sanction was 26.2 (standard deviation [SD] 6.3) years, and the average length of follow‐up was 17.0 (SD 3.9) years in the AAS and 16.6 (SD 4.1) years in the control group (*p* = .031).

The AAS‐group differs significantly statistically from the control group pertaining to educational and occupational status (*p* < .0001). Thus, the control group generally tended to have longer education and a higher degree of employment (see Table 1).

### 3.2 Comparison with the background population

**Figure 1:**
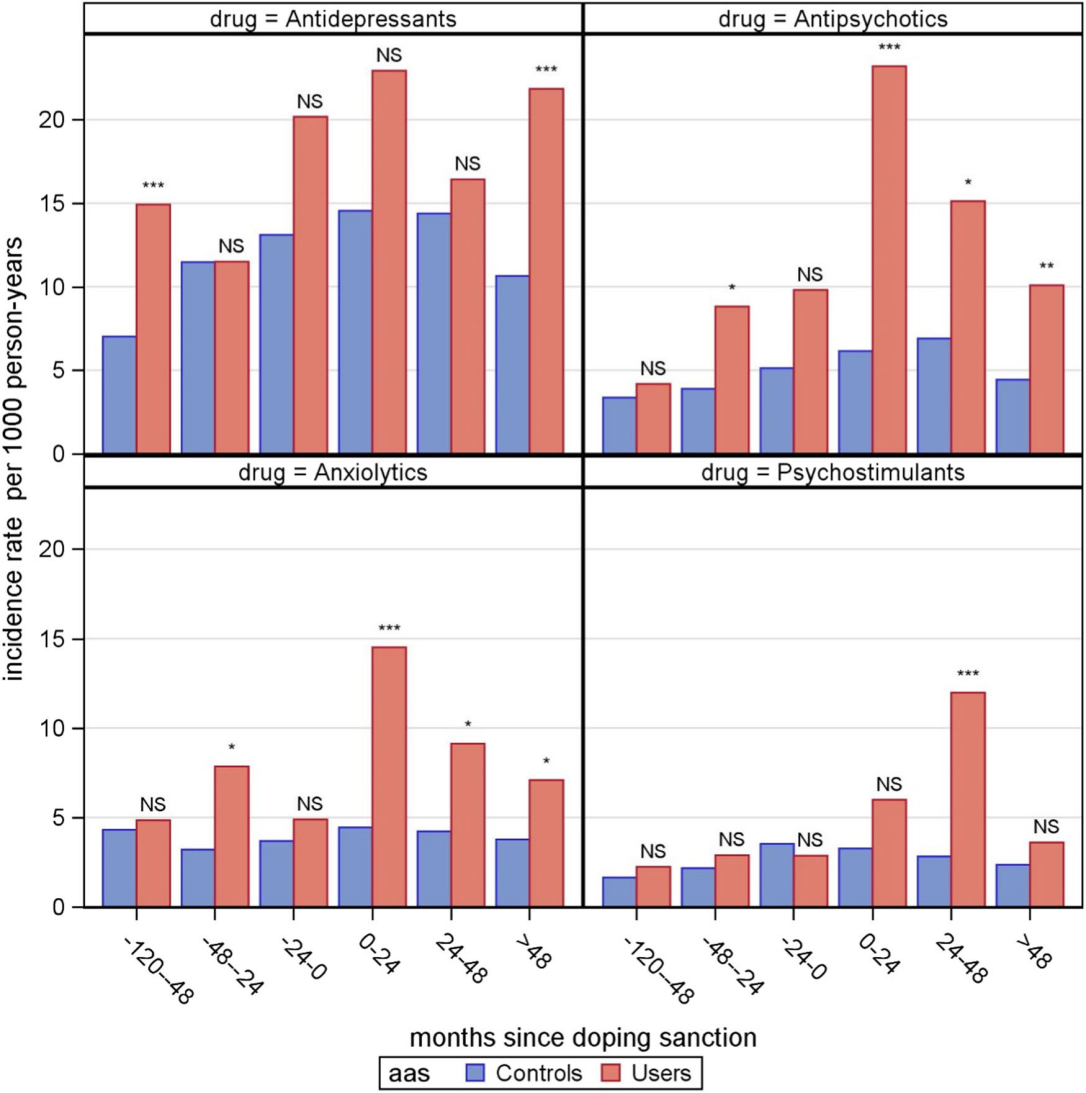
The incidence of psychopharmacological treatment illustrated with incidence rates over time. NS, non significant *p* > .05, *significant *p* = .01−.05, ***p < .001. Data were analyzed with a Poisson regression model.

Table 2 shows the cumulative prevalence of psychopharmacological treatment at baseline and final follow‐up and psychopharmacological treatment is markedly increased in users of AAS. The incidence of psychopharmacological treatment is significantly higher in all four medication groups; antipsychotics (HR: 2.69, 95% CI: 1.99−3.63), anxiolytics (HR: 2.34, 95% CI: 1.62−3.38), antidepressants (HR: 1.65, 95% CI: 1.28−2.13), and psychostimulants (HR: 2.29 95% CI: 1.47−3.57). Furthermore, the incidence of treatment with anxiolytics and antipsychotics is seen to remarkably increase in the years following doping sanction, whereas the incidence of antidepressants use is already markedly elevated several years before doping sanction. Finally, the use of psychostimulants (medication against attention deficit hyperactivity disorder) seems to top 2 years after doping sanction (Figure 1). Table 3 summarizes the prevalence of chronic psychopharmacological treatment and psychiatric diagnoses. Chronic psychopharmacological treatment is significantly more common among AAS users with regard to anxiolytics and antipsychotics. However, the association with antidepressants is only statistically significant in an unadjusted model.

**Table 2:** Incidence of psychopharmacological treatment

	Baseline		Total					
	AAS users (%)	Control (%)	AAS users	Control	HR	*p* Value	HR adjusted	*p* Value
At least one prescription of antipsychotics	5.9	3.5	15.6	7.3	2.69 (1.99−3.63)	<.0001^a^	2.22 (1.63−3.03)	<.0001^a^
At least one prescription of anxiolytics	5.1	3.6	11.6	6.4	2.34 (1.62−3.38)	<.0001^a^	2.27 (1.56−3.31)	<.0001^a^
At least one prescription of antidepressants	13.8	8.3	26.2	16.3	1.65 (1.28−2.13)	.0001^a^	1.44 (1.11−1.87)	.006^a^
At least one prescription of psychostimulants	2.4	2.0	6.8	3.9	2.29 (1.47−3.57)	.0002^a^	1.86 (1.19−2.93)	.007^a^

*Note*: Baseline: The cumulative prevalence at baseline, total: The cumulative prevalence for the entire period of investigation (baseline plus follow‐up).

Abbreviation: AAS, anabolic androgenic steroid.

^a^ Significant *p* value. Data were analyzed with time to event analysis (a Cox proportional hazard regression model).

**Table 3:** Chronic psychopharmacological treatment and diagnoses

	AAS (%)	Control (%)	OR (unadjusted)	*p* Value	OR (adjusted)	*p* Value
Medication						
Five or more prescriptions of antipsychotics (N05A)	6.6	3.5	1.94 (1.34−2.80)	.0004^a^	1.71 (1.15−2.55)	.008^a^
Five or more prescriptions of anxiolytics (N05B)	3.3	1.4	2.35 (1.40−3.96)	.001^a^	2.1 (1.19−3.70)	.01^a^
Five or more prescriptions of antidepressants (N06A)	12.5	9.2	1.41 (1.08−1.85)	.01^a^	1.28 (0.96−1.70)	.09
Five or more prescriptions of psychostimulants (N06B)	4.0	2.9	1.43 (0.91−2.25)	.13	1.08 (0.67−1.75)	.74
Diagnoses						
F00−F09 Organic, including symptomatic, mental disorders	NA	NA	2.00 (0.44−9.17)	.37	2.13 (0.44−10.43)	.35
F10−F19 Mental and behavioral disorders due to psychoactive substance use	5.1	2.2	2.41 (1.58−3.67)	<.0001^a^	1.93 (1.24−2.99)	.004^a^
F20−F29 Schizophrenia, schizotypal and delusional disorders	1.7	1.9	0.85 (0.43−1.70)	.65	0.74 (0.36−1.51)	.40
F30−F39 Mood [affective] disorders	3.3	3.1	1.08 (0.66−1.77)	.76	0.9 (0.54−1.52)	.71
F40−F48 Neurotic, stress‐related, and somatoform disorders	7.5	5.5	1.41 (1.00−1.97)	.048^a^	1.13 (0.80−1.61)	.49
F50−F59 Behavioral syndromes associated with physiological disturbances and physical factors	NA	NA	2.74 (0.76−9.84)	.12	3.84 (1.00−14.69)	.05^a^
F60−F69 Disorders of adult personality and behavior	1.3	1.7	0.75 (0.35−1.62)	.47	0.62 (0.28−1.37)	.24
F70−F79 Mental retardation	NA	NA	0.53 (0.07−3.93)	.53	0.37 (0.05−3.02)	.35
F80−F89 Disorder of psychological development	NA	NA	0.45 (0.11−1.87)	.27	0.38 (0.09−1.60)	.19
F90−F99 Behavioral and emotional disorders with onset usually occurring in childhood and adolescence	5.0	3.2	1.59 (1.05−2.41)	.029^a^	1.22 (0.79−1.89)	.38
Any psychiatric hospital contact	18.0	13.5	1.41 (1.12−1.78)	.004^a^	1.15 (0.9−1.48)	.27

Abbreviations: AAS, anabolic androgenic steroid; NA, not applicable.

^a^ Significant *p* value. Data were analyzed with a logistic regression.

More formal psychiatric diagnoses, especially the prevalence of “Mental and behavioral disorders due to psychoactive substance use,” appear to be strongly associated with AAS use. There was not a higher prevalence of schizophrenia among the AAS‐users as compared to controls.

### 3.3 Self‐controlled observational design

Finally, we analyzed the incidence of psychopharmacological treatment 2 years before doping with that of 2 years following doping sanction. We hereby found that the incidence of antipsychotic and anxiolytic treatment was significantly higher in the period following doping sanction. Thus, the risk of initiating antipsychotic treatment was 2.36 times higher (RR: 2.36, 95% CI: [1.12−4.99], *p* = .024) and the risk of initiating anxiolytic treatment was 2.97 times higher (RR: 2.97, 95% CI: [1.07−8.24], *p* = .037) than in the 2 years period before doping sanction.

The incidence of psychostimulant treatment (RR = 2.10, 95% CI: [0.52−8.36], *p* = .30) and treatment with antidepressants (RR = 1.14, 95% CI: [0.61−2.13], *p* = .30) showed a nonsignificant trend (see Figure 1).

### 3.4 Replication cohort

Data on the replication cohort is found in the Supporting Information material. Overall, the data corroborates our findings that although the use of psychopharmacological treatment was significantly higher among doping sanctioned compared to controls even years before doping sanction, and they generally displayed a higher degree of psychiatric morbidity (Supporting Information: Tables S2 and S3).

## 4 DISCUSSION

In this retrospective study, we analyzed the association between use of AAS and psychopharmacological treatment and psychiatric diagnoses. We found a significant association between AAS and use of antipsychotics, anxiolytics, antidepressants, and psychostimulants, but surprisingly we did not find an increased hospitalization due to psychiatric disorders (including diagnoses of mood disorders). Probably indicating that most of these psychiatric side effects are relatively mild. In this context, it is worth noting that disorders exclusively treated in General Practice in Denmark, that is, outside the hospital system, are not available for research purposes. Therefore, we used medical treatment as a proxy for psychiatric disorders.

Several other clinical and observational studies underline the associations between AAS use and neuropsychiatric symptoms (Havnes et al., 2019; Horwitz & Christoffersen, 2019; Perry et al., 2003; Pope et al., 2000; Thiblin & Pärlklo, 2002).

Already in the late 1980's, Pope and Katz (1988) conducted a smaller interview‐based study in 41 athletes who had previously used AAS. In this study, nine subjects (22%) met the diagnostic criteria for a manic or depressive episode during/or after withdrawal from AAS use while five subjects (12%) had experienced psychotics symptoms.

Later, Su et al. (1993) investigated the acute neuropsychiatric effects of AAS in a placebo‐controlled study including 20 healthy male volunteers in sequential 3 days trials of high‐ and low dose testosterone, respectively. They found that high doses of AAS increased self‐confidence, forgetfulness, distractibility, mood swings, and violent feelings. Recently, Havnes et al. also found that 78% of 232 AAS users reported physical and mental problems, whereas 13.4% experienced physical adverse effects alone. The most common physical adverse effects were anxiety, behavioral change, and depression (Havnes et al., 2019).

Smit et al. followed 100 male volunteers in the Haarlem‐study, and assessed their mental well‐being before, during and after an AAS cycle using psychological questionnaires. They found a prevalence of mild to moderate depression before a cycle of 10%, and this figure did not vary significantly throughout the total cycle (Smit et al., 2021).

Our data displayed interesting temporal trends regarding initiation of anxiolytic and antipsychotic treatment in the years following doping sanction. In contrast, the incidence of antidepressive treatment was markedly elevated throughout the entire period of investigation. Hence can these findings be seen as a consequence of AAS use, hypogonadism, or a matter of confounding?

AAS have a profound impact on the hypothalamic‐pituitary‐testicular axis, and recovery after cessation of AAS use has been reported to have a duration of 3−18 months and may only be partial (Botman et al., 2022; Handelsman, 2021; Smit et al., 2021).

Thus, the high incidence of treatment with anxiolytics and antipsychotics in the year following doping sanction is plausible to compensate for the deprived amount of testosterone. Low levels of testosterone is known to have an adverse impact on mental health and treatment with testosterone is known to alleviate depression in men (Walther et al., 2019).

Studies on animals have shown that anxiety and aggression are elevated after chronic exposure to AAS. These effects are to some extent explained through a complex interaction in the neuronal network which are believed to generate anxiety and aggression. Studies have demonstrated alterations of classical neurotransmitters, such as GABA in relation to anxiety, and neuromodulatory peptides, such as arginine vasopressin and substance P in relation to aggression (Oberlander & Henderson, 2012).

Altogether, evidence supports that AAS have detrimental mental health effects, the mechanism might be mediated both through low levels of testosterone in the abstinence period and through direct neuromodulatory effects. We found a high incidence of psychopharmacological treatment in the years following doping sanction, and this may indicate that withdrawal of AAS and possibly rather immediate onset of altered androgen hormonal status is associated with psychological adverse effects.

However, the current study is not without limitation. First, this study builds on the hypothesis that psychopharmacological treatment is a proxy of psychiatric illness. Second, the doping sanction in itself might precipitate psychiatric adverse effects and the isolated effect of sanction and the potential stigma associated with this or lack of exercise may also have adverse mental health effects. Third, it is well‐known that users of AAS have a hazardous lifestyle and illicit drug abuse is common (Christoffersen et al., 2019; Sagoe et al., 2015). Fourth, we believe our results are associated with a high internal validity. However, our study was only performed in Denmark and this may potentially influence the external validity. Fifth, testing was not done randomly, and some AAS users may not have the bodybuilder physique and hence not been a target for doping control. Finally, the amount and duration of the exposure are missing, and it should be noted, that the extent of which a positive test led to a cessation or a reduction in androgen use cannot be verified. A large fraction of these men probably continued to use androgens. In contrast, the major strength of this study is the relatively large sample size, and the nationwide high‐quality registries, which allows us to follow an individual continuously over time. Additionally, it seems unlikely that a person with severe depression will administer AAS and perform extensive training, and this may contribute to a certain degree of selection bias in the direction opposite of our findings.

## 5 CONCLUSION

In this large nationwide cohort, we found that AAS use is strongly associated with psychopharmacological treatment, especially in the years following doping sanction, indicating treatment of psychiatric disorders. However, we did not find an increased hospitalization due to psychiatric disorders. These findings could not alone be attributed to socioeconomic factors, and this study seems to corroborate the current knowledge of AAS and mental health and might implicate acute withdrawal of AAS as a possible mechanism.

## ETHICS STATEMENT

This study was approved by the Danish Data Protection Agency (2012‐58‐0004/BFH‐2017‐105/05949) and the Danish National Board of Health (FSEID‐00003570/FSEID‐00004621).

## Supporting information

Supporting information.

## Data Availability

Data sharing is not applicable to this article as no new data were created or analyzed in this study.

## References

[da23287-bib-0001] Amaral, J. , Deslandes, A. C. , Padilha, M. C. , Vieira Neto, L. , Osorio, L. E. , Aquino Neto, F. R. , & Cruz, M. S. (2022). No association between psychiatric symptoms and doses of anabolic steroids in a cohort of male and female bodybuilders. Drug Testing and Analysis, 14(6), 1079–1088. 35092181 10.1002/dta.3230 PMC9303351

[da23287-bib-0002] Bjørnebekk, A. , Walhovd, K. B. , Jørstad, M. L. , Due‐Tønnessen, P. , Hullstein, I. R. , & Fjell, A. M. (2017). Structural brain imaging of long‐term anabolic‐androgenic steroid users and nonusing weightlifters. Biological Psychiatry, 82, 294–302. 27616036 10.1016/j.biopsych.2016.06.017

[da23287-bib-0003] Botman, E. , Smit, D. L. , & de Ronde, W. (2022). Clinical question: How to manage symptoms of hypogonadism in patients after androgen abuse? Clinical Endocrinology. Advance online publication. 10.1111/cen.14686 35133022

[da23287-bib-0004] Christoffersen, T. , Andersen, J. T. , Dalhoff, K. P. , & Horwitz, H. (2019). Anabolic‐androgenic steroids and the risk of imprisonment. Drug and Alcohol Dependence, 203, 92–97. 31421475 10.1016/j.drugalcdep.2019.04.041

[da23287-bib-0005] Fischer, S. , Ehlert, U. , & Amiel Castro, R. (2019). Hormones of the hypothalamic‐pituitary‐gonadal (HPG) axis in male depressive disorders—A systematic review and meta‐analysis. Frontiers in Neuroendocrinology, 55, 100792. 31557486 10.1016/j.yfrne.2019.100792

[da23287-bib-0006] Handelsman, D. J. (2021). Androgen misuse and abuse. Endocrine Reviews, 42, 457–501. 33484556 10.1210/endrev/bnab001

[da23287-bib-0007] Hartgens, F. , & Kuipers, H. (2004). Effects of androgenic‐anabolic steroids in athletes. Sports Medicine, 34, 513–554. 15248788 10.2165/00007256-200434080-00003

[da23287-bib-0008] Havnes, I. A. , Jørstad, M. L. , & Wisløff, C. (2019). Anabolic‐androgenic steroid users receiving health‐related information: Health problems, motivations to quit and treatment desires. Substance Abuse Treatment, Prevention, and Policy, 14, 20. 31096999 10.1186/s13011-019-0206-5 PMC6524231

[da23287-bib-0009] Horwitz, H. , Andersen, J. T. , & Dalhoff, K. P. (2019). Health consequences of androgenic anabolic steroid use. Journal of Internal Medicine, 285, 333–340. 30460728 10.1111/joim.12850

[da23287-bib-0010] Horwitz, H. , & Christoffersen, T. (2019). A review on the health hazards of anabolic steroids. Adverse Drug Reaction Bulletin, 317, 1227–1230.

[da23287-bib-0011] Kaufman, M. J. , Janes, A. C. , Hudson, J. I. , Brennan, B. P. , Kanayama, G. , Kerrigan, A. R. , Jensen, J. E. , & Pope, H. G. (2015). Brain and cognition abnormalities in long‐term anabolic‐androgenic steroid users. Drug and Alcohol Dependence, 152, 47–56. 25986964 10.1016/j.drugalcdep.2015.04.023 PMC4458166

[da23287-bib-0012] McHenry, J. , Carrier, N. , Hull, E. , & Kabbaj, M. (2014). Sex differences in anxiety and depression: Role of testosterone. Frontiers in Neuroendocrinology, 35, 42–57. 24076484 10.1016/j.yfrne.2013.09.001 PMC3946856

[da23287-bib-0013] Nackeeran, S. , Patel, M. S. , Nallakumar, D. T. , Ory, J. , Kohn, T. , Deibert, C. M. , Carto, C. , & Ramasamy, R. (2022). Testosterone therapy is associated with depression, suicidality, and intentional self‐harm: Analysis of a national federated database. The Journal of Sexual Medicine, 19, 933–939. 35437187 10.1016/j.jsxm.2022.03.611

[da23287-bib-0014] Nead, K. T. (2019). Androgens and depression: A review and update. Current Opinion in Endocrinology, Diabetes & Obesity, 26, 175–179. 30958398 10.1097/MED.0000000000000477

[da23287-bib-0015] Oberlander, J. G. , & Henderson, L. P. (2012). The Sturm und Drang of anabolic steroid use: Angst, anxiety, and aggression. Trends in Neurosciences, 35, 382–392. 22516619 10.1016/j.tins.2012.03.001 PMC4127319

[da23287-bib-0016] Perry, P. J. , Kutscher, E. C. , Lund, B. C. , Yates, W. R. , Holman, T. L. , & Demers, L. (2003). Measures of aggression and mood changes in male weightlifters with and without androgenic anabolic steroid use. Journal of Forensic Sciences, 48, 2002240. 12762541

[da23287-bib-0017] Pope, H. G. , & Katz, D. L. (1988). Affective and psychotic symptoms associated with anabolic steroid use. American Journal of Psychiatry, 145, 487–490. 3279830 10.1176/ajp.145.4.487

[da23287-bib-0018] Pope, H. G. , Kouri, E. M. , & Hudson, J. I. (2000). Effects of supraphysiologic doses of testosterone on mood and aggression in normal men: A randomized controlled trial. Archives of General Psychiatry, 57, 133–140. Discussion 155−156. 10665615 10.1001/archpsyc.57.2.133

[da23287-bib-0019] Pope, H. G. , Wood, R. I. , Rogol, A. , Nyberg, F. , Bowers, L. , & Bhasin, S. (2014). Adverse health consequences of performance‐enhancing drugs: An Endocrine Society scientific statement. Endocrine Reviews, 35, 341–375. 24423981 10.1210/er.2013-1058 PMC4026349

[da23287-bib-0020] Rahnema, C. D. , Lipshultz, L. I. , Crosnoe, L. E. , Kovac, J. R. , & Kim, E. D. (2014). Anabolic steroid‐induced hypogonadism: Diagnosis and treatment. Fertility and Sterility, 101, 1271–1279. 24636400 10.1016/j.fertnstert.2014.02.002

[da23287-bib-0021] Rasmussen, J. J. , Selmer, C. , Østergren, P. B. , Pedersen, K. B. , Schou, M. , Gustafsson, F. , Faber, J. , Juul, A. , & Kistorp, C. (2016). Former abusers of anabolic androgenic steroids exhibit decreased testosterone levels and hypogonadal symptoms years after cessation: A case‐control study. PLoS One, 11, e0161208. 27532478 10.1371/journal.pone.0161208 PMC4988681

[da23287-bib-0022] Sagoe, D. , McVeigh, J. , Bjørnebekk, A. , Essilfie, M. S. , Andreassen, C. S. , & Pallesen, S. (2015). Polypharmacy among anabolic‐androgenic steroid users: A descriptive metasynthesis. Substance Abuse Treatment, Prevention, and Policy, 10, 12. 25888931 10.1186/s13011-015-0006-5 PMC4377045

[da23287-bib-0023] Sagoe, D. , Molde, H. , Andreassen, C. S. , Torsheim, T. , & Pallesen, S. (2014). The global epidemiology of anabolic‐androgenic steroid use: A meta‐analysis and meta‐regression analysis. Annals of Epidemiology, 24, 383–398. 24582699 10.1016/j.annepidem.2014.01.009

[da23287-bib-0024] Sell, A. , Lukazsweski, A. W. , & Townsley, M. (2017). Cues of upper body strength account for most of the variance in men's bodily attractiveness [Internet]. *Proceedings of the Royal Society of Biological Science*, 284(1869), 20171819. 10.1098/rspb.2017.1819 PMC5745404 29237852

[da23287-bib-0025] Smit, D. L. , Buijs, M. M. , Hon, O. , Heijer, M. , & Ronde, W. (2021). Positive and negative side effects of androgen abuse. The HAARLEM study: A one‐year prospective cohort study in 100 men. Scandinavian Journal of Medicine & Science in Sports, 31, 427–438. 33038020 10.1111/sms.13843

[da23287-bib-0026] Su, T. P. , Pagliaro, M. , Schmidt, P. J. , Pickar, D. , Wolkowitz, O. , & Rubinow, D. R. (1993). Neuropsychiatric effects of anabolic steroids in male normal volunteers. JAMA: The Journal of the American Medical Association, 269, 2760–2764. 8492402

[da23287-bib-0027] Thiblin, I. , & Pärlklo, T. (2002). Anabolic androgenic steroids and violence. Acta Psychiatrica Scandinavica, 106, 125–128. 10.1034/j.1600-0447.106.s412.27.x 12072143

[da23287-bib-0028] Walther, A. , Breidenstein, J. , & Miller, R. (2019). Association of testosterone treatment with alleviation of depressive symptoms in men: A systematic review and meta‐analysis. JAMA Psychiatry, 76, 31. 30427999 10.1001/jamapsychiatry.2018.2734 PMC6583468

[da23287-bib-0029] Windfeld‐Mathiasen, J. , Dalhoff, K. P. , Andersen, J. T. , Klemp, M. , Horwitz, A. , & Horwitz, H. (2021). Male fertility before and after androgen abuse. The Journal of Clinical Endocrinology & Metabolism, 106, 442–449. 33196845 10.1210/clinem/dgaa837

