# Supplementary material for: Psychiatric morbidity among men using anabolic steroids
Source: Depress Anxiety. 2022 Oct 25;39(12):805–12. doi: 10.1002/da.23287 (PMC10092709; doi:10.1002/da.23287)
Supplement: Supplementary file 1 — Supporting information. [file DA-39-805-s001.docx]

Supplementary

**Supplementary** **Methods:**

In this section we describe the data used in the analysis based on information from the following Danish registries.

Registries:

The Danish Civil Registration System holds current and historical information on all persons living in Denmark and includes gender, place and date of birth, date of death, place of residence, date of emigration, immigration or disappearance, and information about parents, siblings and spouses (1).

The National Hospital Register contains information about all hospitalizations in the country since 1978, including admittance data and discharge diagnosis and, since 1993, information about outpatient visits. It holds records of more than 99% of all hospital contacts from all Danish hospitals (2). The Danish Psychiatric Central Research Registry contains information on all psychiatric hospital contacts in Demark(3). We used diagnoses of mental disorders up to ten year prior to baseline.

The Danish National Prescription Register holds individual-level data on all prescribed drugs dispensed at all pharmacies in Denmark since 1995. Pharmacies are required by law to register prescriptions digitally which ensures highly accurate prescription data, and completeness has been estimated to be 97.5% (4). We searched for patients with prescriptions of psychiatric medication from ten years prior to baseline and until the end of follow-up.

The DREAM database was established in 1991 and includes information on all Danish residents, who have received social benefits (transfer income) and furthermore it covers data on employment status. The employment status at baseline was divided into four categories: self-supporting (working), short-term sickness (sickness benefits), long-term sickness or disability (including disability pension, Flex-jobs (long-term disabled with partial working capacity)), unemployment benefits (social security benefits). (5,6)

Statistics Denmark holds information on the country of origin and the educational level of all subjects. The following three categories are available according to origin: (i) Persons of Danish origin,

(ii) immigrants, and (iii) descendants of immigrants.(6) The Danish Education Registers contains information about the highest attained level of education of all subjects(7)**.** The level of education at baseline was divided into four groups based on length of education: (i) 10 years or less, (ii) 10–12 years, (iii) 12–15 years, or (iv) 15 years or more.

**Supplementary** **Results:**

**Replication cohort:**

In this section we show data on the replication cohort data (*n*=644 males). This cohort is all males who were sanctioned because they declined to deliver a urine sample and therefor is not verified as androgen users. There is a substantial suspicion that the persons in the replication cohort were using androgens, since they were informed that refusing to participate in the doping control would lead to a doping sanction, and secondly we have previously shown that they present the same socio-demographic profile and prevalence of adverse effects as the cohort of men with laboratory confirmed androgen abuse.

|  | |  | |
| --- | --- | --- | --- |
|  |  | **AAS users** | **Control** |
| Age | Mean (SD) | 28.42 (7.24) | 28.39(7.27) |
| Education groups |  | 4.66 | 4.95 |
| Missing | **%** |  |  |
| 10 years or less | **%** | 46.43 | 30.50 |
| 10 to 12 years | **%** | 11.65 | 19.89 |
| 12 to 15 years | **%** | 33.23 | 28.42 |
| 15 years or more | **%** | 4.04 | 16.24 |
| Country of origin |  | 80.75 | 83.54 |
| Danish | **%** |  |  |
| Immigrants | **%** | 13.04 | 13.32 |
| Descendants of immigrants | **%** | 6.21 | 3.14 |
| Occupational status |  | 2.02 | 1.16 |
| Missing | **%** |  |  |
| Self-supporting | **%** | 69.41 | 83.70 |
| Sick leave - temporarily | **%** | 3.88 | 2.10 |
| Disability benefits | **%** | 4.04 | 2.72 |
| Unemployed | **%** | 20.65 | 10.33 |
| Complete data | **%** | 93.63 | 94.35 |
|  |  |  |  |

**Table S1**: Baseline characteristics

|  |  | **Baseline** |  | **Total** |  |  |  |  |  |
| --- | --- | --- | --- | --- | --- | --- | --- | --- | --- |
|  |  | AAS users | Control | AAS users | Control | HR |  | ProbChiSq | HRadjust |
| At least one prescription of antipsychotics | % | 12.58 | 4.35 | 22.52 | 7.05 | 4.09 (3.07-5.45) |  | <.0001* | 2.79 (2.04-3.83) |
| At least one prescription of anxiolytics | % | 9.16 | 3.62 | 14.60 | 5.40 | 3.25 (2.23-4.75) |  | <.0001* | 2.94 (1.98-4.38) |
| At least one prescription of antidepressants | % | 22.05 | 10.40 | 34.63 | 15.57 | 2.95 (2.31-3.76) |  | <.0001* | 2.32 (1.79-3.01) |
| At least one prescription of psychostimulants | % | 7.61 | 2.64 | 11.34 | 4.07 | 2.72 (1.73-4.26) |  | <.0001* | 1.94 (1.2-3.15) |
|  |  |  |  |  |  |  |  |  |  |

**Table S2:** Incidence of psychopharmacological treatment

Baseline: The cumulative prevalence at baseline, total: The cumulative prevalence for the entire period of investigation (baseline plus follow-up). *:significant p-value.


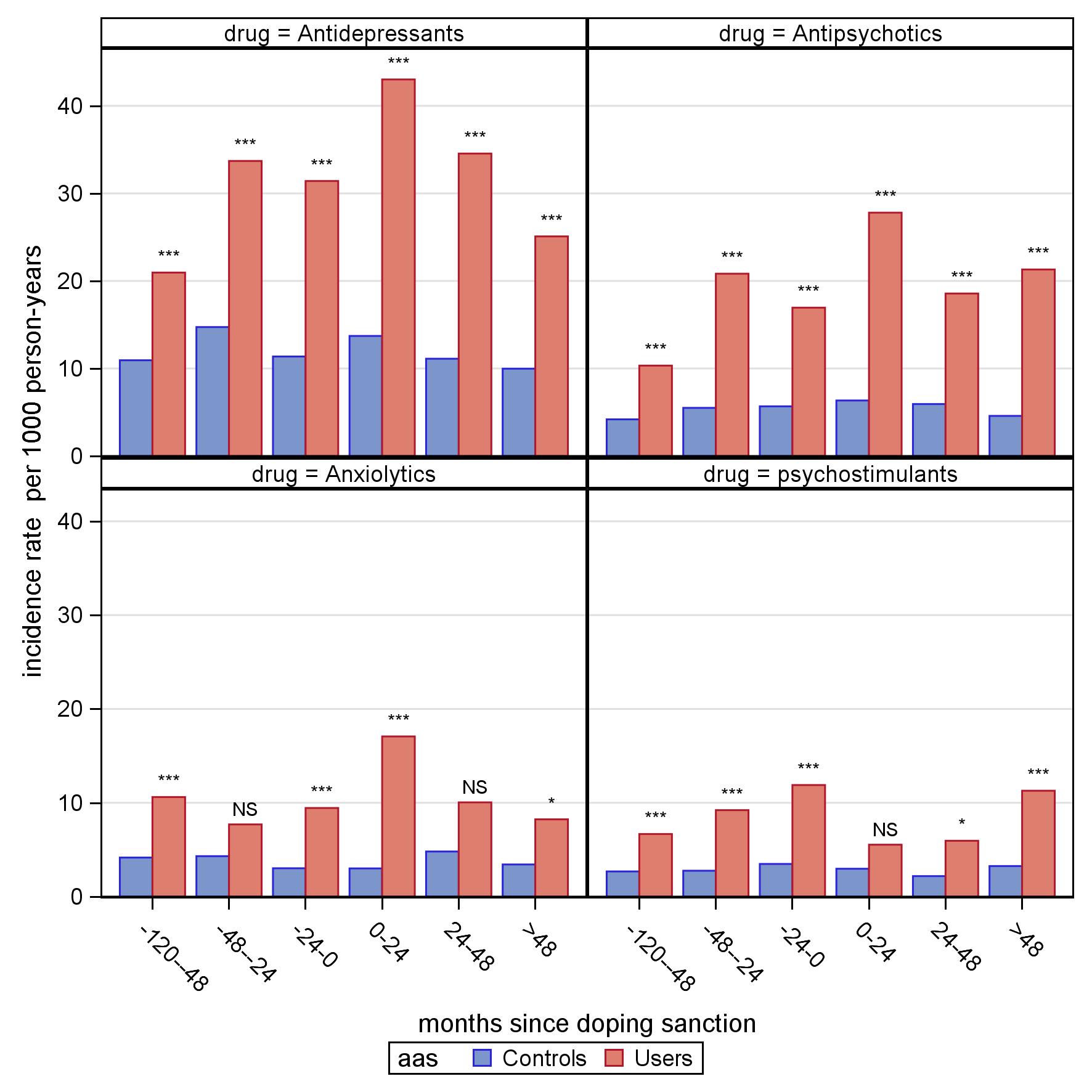


**Figure S1:** The incidence of psychopharmacological treatment illustrated with incidence rates over time. NS: non significant p-value>0.05 , *:significant p-value=0.01-0.05, ***: p<0.001

|  | AAS (%) | Control (%) | OR (unadjusted) | *p*-value | OR (adjusted) | *p*-value |
| --- | --- | --- | --- | --- | --- | --- |
| **Medication** |  |  |  |  |  |  |
| 5 or more prescriptions of antipsychotics (N05A) | 9.5 | 3.5 | 2.88 (2.14-3.87) | <.0001* | 1.8 (1.29-2.52) | 0.0005* |
| 5 or more prescriptions of anxiolytics (N05B) | 3.9 | 1.3 | 3.17 (2.01-5.00) | <.0001* | 1.89 (1.12-3.17) | 0.016* |
| 5 or more prescriptions of antidepressants (N06A) | 18.0 | 8.2 | 2.47 (1.98-3.08) | <.0001* | 1.93 (1.52-2.46) | <.0001* |
| 5 or more prescriptions of psychostimulants (N06B) | 7.9 | 2.9 | 2.88 (2.09-3.96) | <.0001* | 1.95 (1.37-2.77) | 0.0002* |
| **Diagnoses** |  |  |  |  |  |  |
| F00-F09 Organic, including symptomatic, mental disorders | 0.6 | 0.2 | 3.09 (1.00-9.50) | 0,049* | 2.22 (0.68-7.26) | 0.19 |
| F10-F19 Mental and behavioral disorders due to psychoactive substance use | 5.9 | 1.9 | 3.17 (2.18-4.60) | <.0001* | 1.99 (1.32-2.99) | 0.001* |
| F20-F29 Schizophrenia, schizotypal and delusional disorders | 2.2 | 1.8 | 1.21 (0.69-2.12) | 0.50 | 0.78 (0.43-1.43) | 0.42 |
| F30-F39 Mood [affective] disorders | 5.4 | 2.6 | 2.16 (1.49-3.14) | <.0001* | 1.64 (1.1-2.45) | 0.0147* |
| F40-F48 Neurotic, stress-related and somatoform disorders | 13.8 | 5.5 | 2.74 (2.14-3.51) | <.0001* | 2.07 (1.58-2.71) | <.0001* |
| F50-F59 Behavioral syndromes associated with physiological disturbances and physical factors | NA | NA | 1.77 (0.52-6.05) | 0.36 | 2.29 (0.63-8.33) | 0.20 |
| F60-F69 Disorders of adult personality and behavior | 3.57 | 1.54 | 2.37 (1.50-3.76) | 0.0002* | 1.71 (1.05-2.79) | 0.03* |
| F70-F79 Mental retardation | NA | NA | 0.36 (0.05-2.62) | 0.31 | 0.23 (0.03-1.70) | 0.15 |
| F80-F89 Disorder of psychological development | NA | NA | 0.17 (0.02-1.22) | 0.08 | 0.11(0.02-0.84) | 0.03* |
| F90-F99 Behavioral and emotional disorders with onset usually occurring in childhood and adolescence | 9.78 | 2.97 | 3.55 (2.64-4.78) | <.0001* | 2.18(1.56-3.04) | <.0001* |
| Any psychiatric hospital contact | NA | 12.34 | 2.91 (2.41-3.50) | <.0001* | 2.15(1.74-2.65) | <.0001* |

**Table S3**: Chronic psychopharmacological treatment and diagnoses

NA: not applicable, *:significant p-value

**References**

1. Pedersen CB. The Danish Civil Registration System. Scand J Public Health. juli 2011;39(7 Suppl):22–5.

2. Lynge E, Sandegaard JL, Rebolj M. The Danish National Patient Register. Scand J Public Health. juli 2011;39(7 Suppl):30–3.

3. Mors O, Perto GP, Mortensen PB. The Danish Psychiatric Central Research Register. Scand J Public Health. juli 2011;39(7 Suppl):54–7.

4. Sørensen HT, Hansen I, Ejlersen E, Sabroe S, Hamburger H. Identification of patients treated with strong analgesics: an assessment of two Danish information systems with respect to epidemiological research. J Med Syst. februar 1996;20(1):57–65.

5. Horwitz H, Dalhoff KP, Klemp M, Horwitz A, Andersen JT, Jürgens G. The prognosis following amphetamine poisoning. Scand J Public Health. december 2017;45(8):773–81.

6. Christoffersen T, Andersen JT, Dalhoff KP, Horwitz H. Anabolic-androgenic steroids and the risk of imprisonment. Drug Alcohol Depend. 1. oktober 2019;203:92–7.

7. Jensen VM, Rasmussen AW. Danish Education Registers. Scand J Public Health. juli 2011;39(7 Suppl):91–4.
